# Syndromic diarrhea/Tricho-hepato-enteric syndrome

**DOI:** 10.1186/1750-1172-8-5

**Published:** 2013-01-09

**Authors:** Alexandre Fabre, Christine Martinez-Vinson, Olivier Goulet, Catherine Badens

**Affiliations:** 1 UMR_S 910, Inserm-Faculté de Médecine, Aix-Marseille Université, 13385, Marseille, France; 2 AP-HM, Service de Pédiatrie Multidisciplinaire, Hôpital d’Enfants de la Timone, 13385, Marseille, France; 3 AP-HP, Service de Gastroentérologie, Hôpital Robert Debré, 75019, Paris, France; 4 Pediatric Gastroenterology-Hepatology and Nutrition, Reference Center for Rare Digestive Disease, Hôpital Necker-Enfants Malades/AP-HP, 75743, Paris, France; 5 AP-HM, Laboratoire de Génétique Moléculaire, Hôpital d’Enfants de la Timone, 13385, Marseille, France

**Keywords:** SKI COMPLEX, *SKIV2L*, *TTC37*, WDR61, SKI3, SKI2, SKI8, Intractable diarrhea, Syndromic diarrhea, Tricho-hepato-enteric syndrome, Woolly hair

## Abstract

**Abstract:**

Syndromic diarrhea/Tricho-hepato-enteric syndrome (SD/THE) is a rare and severe bowel disorder caused by mutation in *SKIV2L* or in *TTC37*, 2 genes encoding subunits of the putative human SKI complex. The estimated prevalence is 1/1,000,000 births and the transmission is autosomal recessive. The classical form is characterized by 5 clinical signs: intractable diarrhea of infancy beginning in the first month of life, usually leading to failure to thrive and requiring parenteral nutrition; facial dysmorphism characterised by prominent forehead and cheeks, broad nasal root and hypertelorism; hair abnormalities described as woolly and easily removable; immune disorders resulting from defective antibody production; intrauterine growth restriction. The aetiology is a defect in *TTC37*, a TPR containing protein, or in the RNA helicase *SKIV2L*, both constituting the putative human ski complex. The ski complex is a heterotetrameric cofactor of the cytoplasmic RNA exosome which ensures aberrants mRNAs decay. The diagnosis SD/THE is initially based on clinical findings and confirmed by direct sequencing of *TTC37* and *SKIV2L*. Differential diagnosis with the other causes of intractable diarrhea is easily performed by pathologic investigations. During their clinical course, most of the children require parenteral nutrition and often immunoglobulin supplementation. With time, some of them can be weaned off parenteral nutrition and immunoglobulin supplementation. The prognosis depends on the management and is largely related to the occurrence of parenteral nutrition complications or infections. Even with optimal management, most of the children seem to experience failure to thrive and final short stature. Mild mental retardation is observed in half of the cases.

**Abstract in French:**

Les diarrhées syndromiques ou syndrome tricho-hepato-enterique (SD/THE) sont un syndrome rare et sévère dont l’incidence est estimée à 1 cas pour 1 million de naissances et la transmission autosomique récessive. La forme typique associe 5 signes cliniques: une diarrhée grave rebelle nécessitant dans la majorité des cas une nutrition parentérale du fait de la malnutrition, une dysmorphie avec un front large et bombé, une racine du nez large et un hypertélorisme, des anomalies des cheveux qui sont fragiles, cassants, incoiffables et qualifiés de « laineux », un retard de croissance intra utérine et des anomalies de l’immunité à type de déficit en immunoglobuline ou d’absence de réponse aux antigènes vaccinaux. Des anomalies de deux protéines peuvent être à l’origine du syndrome SD/THE: *TTC37*, une protéine à motif TPR et *SKIV2L*, une hélicase à ARN, toutes 2 étant des constituants du complexe SKI humain. Le complexe SKI est un co-facteur de l’exosome cytoplasmique qui assure la dégradation des ARN aberrants ou exogènes. Le diagnostic est d’abord clinique puis confirmé par le séquençage des gènes *TTC37* et *SKIV2L*. Le diagnostic différentiel avec les autres formes de diarrhées intraitables est fait grâce aux analyses anatomopathologiques qui montrent dans les autres formes, des lésions spécifiques. La prise en charge clinique repose sur la nutrition parentérale et la supplémentation en immunoglobuline si nécessaire. Un certain nombre d’enfants peuvent être sevrés de la nutrition parentérale et des supplémentations en immunoglobulines. En cas d’atteinte hépatique, celle-ci peut être sévère et conduire au décès. Même avec une prise en charge optimale, les enfants présentent une petite taille et, dans la moitié des cas, un retard mental modéré.

Disease name/synonyms – Syndromic diarrhea – Phenotypic diarrhea – Tricho-hepato-enteric syndrome – Intractable diarrhea of infancy with facial dysmorphism – Trichorrhexis nodosa and cirrhosis – Neonatal hemochromatosis phenotype with intractable diarrhea and hair abnormalities – Intractable infant diarrhea associated with phenotypic abnormalities and immune deficiency- Syndromatic diarrhea. [ORPHA84064 MIM 222470 and MIM614602]. Possibly chronic diarrhea and skin hyperpigmentation.

## Definition

The classical form of Syndromic Diarrhea/Tricho-hepato-enteric syndrome (SD/THE) associates an intractable diarrhea of infancy with facial dysmorphism, abnormal hair and immune deficiency; intra uterine growth restriction (IUGR) or small for gestational age (SGA) is often present [1]. SD/THE is a disease genetically heterogeneous, phenotypically homogeneous, caused by mutations in *TTC37* (HGNC:23639) or in *SKIV2L* (HGNC:10898), 2 genes encoding co-factors of the putative human SKI complex [2-4]. SD/THE is classified in the group of congenital diarrheal disorders which consists of 4 categories depending on i) the alteration in absorption and transport of nutrients and electrolytes, ii) enteroendocrine cell differentiation, iii) modulation of the intestinal immune response and iv) enterocyte differentiation and polarization [5]. SD/THE is part of the enterocyte alteration diseases which includes also tufting enteropathy and microvillus atrophy disease [5].

## Historical note

First described by Stankler et al. in 1982 [6] and further delineated by Girault et al. in 1994 [7], the definition of the syndrome has been complicated by the description of the tricho-hepato-enteric syndrome as probably distinctive in 1997 [8]. New cases were reported between 2000 and 2007 [9-12] using alternatively one or the other denominations. In 2007, we proposed to group the two syndromes under the same entity [1]. This suggestion was proven relevant with the imputation of *TTC37*[2,3] as causative of 2/3 of SD/THE cases, without clear clinical delineation. More recently, the identification of *SKIV2L* mutations as causing the missing third of SD/THE permitted us to confirm the unity of the syndrome [4].

## Epidemiology

SD/THE is a rare disease. Between 1982 and 2012, 44 cases have been published [2-15]**]**. During the last 20 years, 13 cases have been diagnosed in children born in France [3,4 and personal data] which, according to the annual number of births in France, indicates a prevalence of at least 1/1,000,000. It should be noted that 21 cases out of 44 are from consanguineous families. The prevalence may be underestimated due to the fact that only patients with important gastrointestinal manifestations are referred to specialists. SD/THE seems to be present in all populations. To date, patients have been described in Indian, European and Mediterranean populations (from the UK, Italy, France, Poland, Turkey and North Africa).

## Clinical description

From the review of the literature, 9 clinical signs are associated with SD/THE [2-15]**]**. Three are constant: intractable diarrhea, facial dysmorphism and hair abnormality. Two are very frequent (more than 90%): IUGR and immunodeficiency; 2 are frequent: skin abnormalities and liver disease and 2 are rare: congenital cardiac defects and platelet anomaly.

The following description is based on the 44 reported cases [2-15] and data from 4 cases referred to our laboratory for genetic exploration. For each sign, the number of positives cases out of the total number of patients is indicated between brackets.

Intractable diarrhea (48/48): All the children presented intractable diarrhea of infancy defined as a chronic diarrhea persistent despite an enteral rest. This disease was at first diagnosed among children hospitalised for feeding problems. The onset of diarrhea was variable, from the first day to 32 weeks of life. Most children had parenteral nutrition during the follow up. The pathology analysis of intestinal tract showed normal (7/44) or mild to severe villous atrophy with no specific alterations, especially no sign specific of tufting enteropathy or microvillus inclusion disease. Colitis (16/22) or gastritis has also been found.

Hair abnormalities are one of the most consistent signs as all children presented abnormal hair (48/48). Hair was described most often as woolly, easily removable, unmanageable, brittle and scanty. When searched, trichorrhexis nodosa were frequently found (35/39).

**Figure 1:**
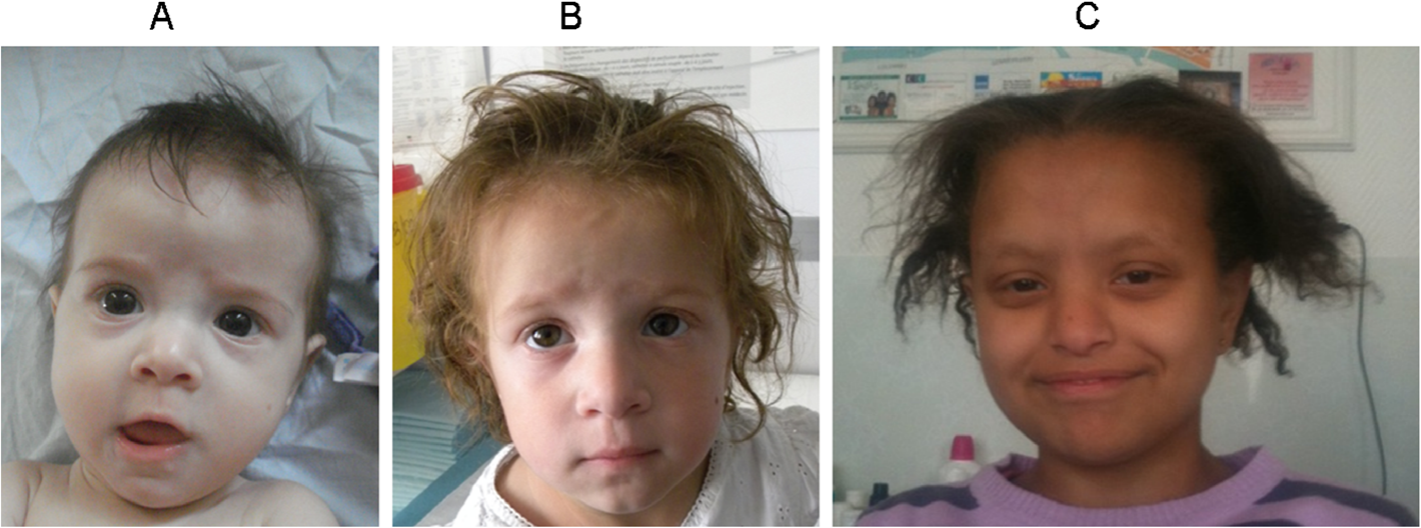
**Clinical presentation of patients with mutations either in *TTC37 *(A at 8 month and B at 3 year) or *SKIV2L *(C).**

Facial dysmorphism (47/47): Children presented wide forehead, broad nasal root, hypertelorism, and coarse features (Figure 1). The facial dysmorphism can become more evident with time.

Immune defect (39/44): this sign was the most difficult to appreciate as investigations were different from one case to another. Signs reported most frequently were a low immunoglobin level (24 patients), a defect in antibody production after vaccination (14), monoclonal hyper IgA (3), a low lymphocytes count (2). Some children needed an immunoglobulin supplementation (temporarily or for a long period), some were prone to frequent infections.

**Figure 2:**
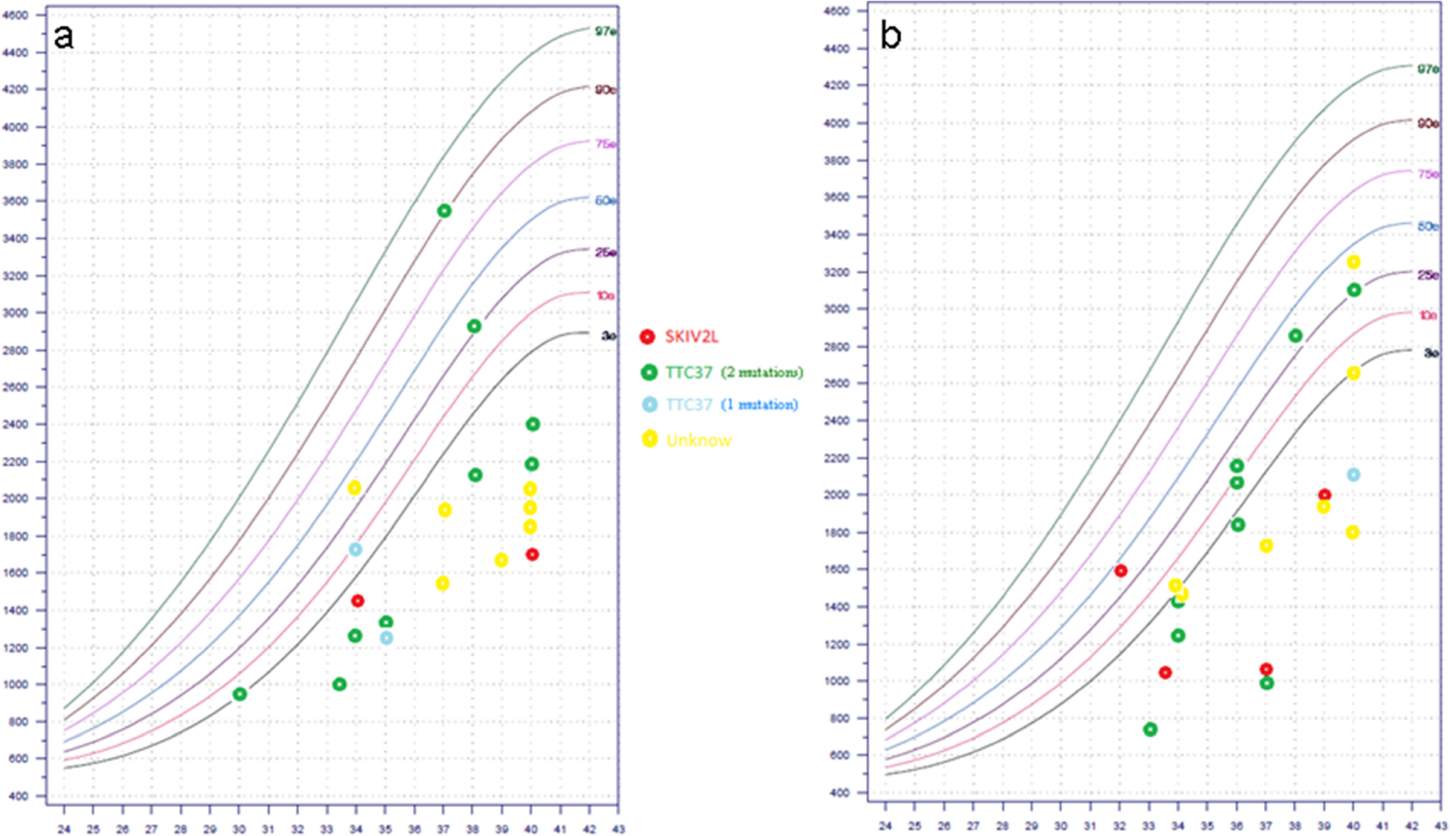
Birth weight for a) baby boys and b) baby girls with syndromic diarrhea/tricho-hepato-enteric syndrome according to molecular defects (Audipog Chart Growht http://www.audipog.net).

IUGR/SGA (31/46): Most children had a small birth weight for gestational age: out of 41 with recorded weight, 30 children were below the 10th percentile and 27 below the third percentile (Figure 2). Moreover, 19 children out of 45 were preterm, due to medical extraction for severe IUGR in several cases.

Liver disease (23/44): More than half of the children had a liver disease. Cirrhosis (18) and siderosis were the main signs. Rarely, patients presented an isolated hepatomegaly. One patient was described as having a hepatoblastoma .

Skin abnormalities (18/36): Half of the children with skin description presented abnormalities (café au lait spot, xerosis, rubbery skin).

Cardiac abnormalities (8/31): Several children presented cardiac abnormalities: aortic insufficiency (2 patients), peripheral pulmonary stenosis (one patient), tetralogy of Fallot (one patient), atrial septal defect or ventricular septal defect (4 patients), persistent arterial duct (1 patient).

Platelet: first described by Hartley et al., platelet morphology has not been investigated in all patients. Increased size of the platelet was found in 5 out of 21 patients and was temporary in some cases. Phenotypic abnormalities observed in transmission electron microscopy were not performed in studies other than that of Hartley et al.

Other signs (inguinal hernia, thymus atrophy, small kidneys, Perthe syndrome, glaucoma, hypothyroid, polycystic kidney) were described in only a few patients. The association with THE syndrome might be coincidental.

Finally a slight mental retardation is noted in more than half of the patients but this is not well documented.

Signs described in THE syndrome are summarised in Table 1 with corresponding mutations. Regarding the data currently available, no genotype/phenotype correlation can be made either with the causative gene (*TTC37* or *SKIV2L*) or the mutation's type. In the case of children bearing the same mutation, the phenotype severity can vary from one individual to another.

**Table 1:** Summary of clinical signs according to molecular defects

	**All (n = 48)**	**Patients with 2 mutations in *TTC37 *(n = 22)**	**Patient with 1 mutation in *TTC37 *(n = 3)**	**Patient with 2 mutations in *SKIV2L *(n = 7)**	**Patients not tested (n = 16)**	**Patient from Alqoed (n = 7)**	**Patient with mutation p.Trp936X (n = 5)**
**Consanguineous**	21/44	14/21	0/3	4/7	3/13	6/7	5/5
**Born before 37 we**	19/45	11/21	2/3	3/6	3/15	1/7	2/5
**Sex (F/M)**	27/20	13/9	1/2	5/2	8/7	4/3	3/2
**Birthweight mean**	1825 (780–3.580)	1906 (780–3580)	1715 (1345–2100)	1473 (1010–2000)	1896 (1410–3250)	2.61 (1.6-3.9)	1986 (1375–2400)
**Intractable diarrhea**	48/48	22/22	3/3	7/7	16/16	7/7	5/5
**Onset of diarrhoea (weeks) means**	5.25 (1–32)	6.8 (1–32)	4	3.7 (1–4)	4.9 (1–24)	2 (1–5.7)	
**Facial dysmorphy**	47/47	22/22	3/3	6/6	16/16	6/7	5/5
**Hair abnormalities**	48/48	22/22	3/3	7/7	16/16	6/7	5/5
**Trichorhexis nodosa**	35/39	18/19	2/3	6/6	9/11	2/5	4/4
**Immune deficiency**	39/44	19/20	3/3	4/7	13/14	0/7	4/5
**IUGR/SGA**	31/46	12/21	2/3	5/7	12/15	5/7	5/5
**Liver disease**	23/44	11/19	0/3	3/6	9/16	2/7	2/3
**Skin abnormalities**	18/36	9/19	0/2	¾	6/11	7/7	2/5
**Platelet abnormalities**	5/21	5/17	0/2	0/2			2/3
**Cardiac abnormalities**	8/31	5/18	1/2	1/3	1/8		2/5
**Outcome (died/alive)**	18/30	5/17	0/3	2/5	11/5		3/2

## Aetiology

SD/THE is an autosomal recessive disease caused by mutation in *SKIV2L* in 40% of the cases or *TTC37* in 60% of the cases [2-4]. These genes encode proteins ortholog of the yeast Ski2p and Ski3p respectively. In the yeast, ski2p and ski3p form, with two copies of ski8p, the ski complex [16]; it is thus likely that *SKIV2L* and *TTC37* form along with WDR61, the human ortholog of Ski8p, the putative human SKI complex. The functional part of the complex is *SKIV2L* which is an RNA helicase, *TTC37* and WDR61 (containing respectively TPR and WDR repeats) being involved in protein-protein interactions. In 2005, Zhu et al. showed that these 3 human proteins co-localized with the human PAF but their association within a functional complex remains to be formally demonstrated [17]. The precise function of the ski complex in human is unknown but the ortholog genes are conserved throughout evolution in all eukaryotes especially in yeast and fruit fly, two models which provide most of the current data [18]. The ski complex is the cofactor of the cytosolic exosome whose function is to decay aberrant mRNAs in the 3’-5’ way. The targeted mRNAs are no go, no stop mRNA and viral RNA. The ski complex is essential to this function but the absence of a functional ski complex is not lethal probably because of the existence of the other mRNA decay pathway in the sense 5’-3’, which is mediated by XRN1.

Most mutations are private mutations and are distributed in all exons without hot spot either in *TTC37* or in *SKIV2L*. For *TTC37*, among 25 mutations found, 6 are missense, 5 are stop mutations, 5 are frame shift mutations due to deletion or insertion within the coding region and 9 are mutations in splice sites (3 of which cause a frame shift, 1 a deletion in the protein, 1 both a deletion and an insertion, and 4 have unknown effects on the protein). Trp936* is one of the rare recurrent mutations and has been found in 4 families among 25, all originating from India or Pakistan. For *SKIV2L*, among 9 mutations explored in 7 individuals from 7 families, 5 are deletions or insertion leading to frame shift, 3 are stop mutations and 1 a missense. There is no recurrent mutation. Table 2 summarizes currently published mutations.

**Table 2:** **Summary of mutations identified in *TTC37 *and *SKIV2L***

**Gene**	**Type of mutation**	**Mutation (Transcript)**	**Mutation (Protein)**	**Exon**	**Number of families**	**Number of affected individuals**	**References for mutation description**	**References for clinical description**
*TTC37*	Deletion	c.287_291del	p.(Leu96Trpfs*11)	6	1	2	[3]	[15] patient 22
	Stop	c.439C < T	p.(Gln147*)	8	1	1	[2]	[2] patient 10
	Missense	c.751G < A	p.(Phe215GluFs*14)	10	1	1	[2]	[2] patient 7
	Deletion	c.811del	p.(Ser271Valfs*8)	11	1	1	Personal Data	
	Deletion	c.1168del	p.(Val390Phefs*30)	14	1	1	[3]	
	Deletion	c.1300_1301del	p.(Lys434Glyfs*14)	15	1	1	[2]	[2] patient 9
	Duplication	c.1305dup	p.(Tyr436Leufs*13)	15	1	1	Personal Data	
	Splice site	c.1632 + 1del	p.(Glu545Phefs*40)	17	1	1	[2] patient 5	[2]
	Splice site	c.1453-1G < C	ND	17	1	1	Personal Data	
	Stop	c.1708C < T	p.(Arg570*)	18	1	1	Personal Data	
	Stop	c.2251C < T	p.(Gln751*)	21	1	1	[2]	[2] patient 10
	Splice site	c.2515 + 1G < C	p.(Cys813ValfsX56)	23	1	1	[3]	
	Splice site	c.2578-7_2578-3del	p.(Asn860_878GluDel)	25	1	1	[3]	[1] patient 1
	Splice site	c.2779-2A < G	p.(Glu974Glyfs*19)	28	2	2	[2]	[2] patients 4 and 6
	Stop	c.2808G < A	p.(Trp936*)	28	4	5	[2]	[2] patients 1,2, 8,12,
	Splice site	c.2921-2G < A	ND	29	1	1	[14]	
	Splice site	c.3015-1G < A	ND	30	1	1	[3]	
	Splice site	c.3564-2A < G	ND	31	1	1	[3]	
	Missense	c.3230C < A	p.(Ala1077Asp)	32	1	1	[3]	[15] patient 18
	Missense	c.3808C < G	p.(Pro1270Ala)	37	1	1	[3]	
	Missense	c.3847G < A	p.(Asp1283Asn)	37	2	2	[2]	[2] patients 3 and 11
	Stop	c.3960C < A	p.(Tyr1320*)	38	1	1	[3]	[1] patient 2
	Missense	c.4454T < G	p.(Leu1485Arg)	41	1	1	[3]	
	Missense	c.4514T < C	p.(Leu1505Ser)	42	2	2	[2]	[2] patient 9
	Splice site	c.4620 + 1G < C	p.(Trp1524_1564DelIns61)	42	1	1	[3]	[1] patient 1
*SKIV2L*	Stop	c.848G < A,	p.(Trp283*)	9	1	1	[4]	
	Missense	c.1022T < G	p.(Val341Gly)	10	1	1	[4]	
	Deletion	c.1434del	p.(Ser479Alafs*3)	14	1	1	[4]	[15] patient 25
	Insertion	c.1635_1636insA,	p.(Gly546Argfs*35)	15	1	1	[4]	[15] patient 21
	Stop	c.2266C < T	p.(Arg756*)	19	1	1	[4]	[15] patient 23
	Stop	c.2442G < A	p.(Trp814*)	20	1	1	[4]	[15] patient 23
	Deletion	c.2572del	p.(Val858*)	21	1	1	[4]	
	Deletion	c.2662_2663del	p.(Arg888Glyfs*12)	22	1	1	[4]	
	Deletion	c.3561_3581del	p.(Ser1189_Leu1195del)	28	1	1	Personal Data	

## Diagnosis and diagnostic methods

The association of intractable diarrhea of infancy, i.e. chronic diarrhea persisting despite enteral rest, with woolly hair and dysmorphism is highly suggestive of SD/THE. The analysis of an intestinal biopsy is indicated to rule out other forms of intractable diarrhea caused by enterocyte abnormalities. The diagnosis is confirmed by the sequencing of *TTC37* and *SKIV2L*.

## Differential diagnosis

SD/THE are mostly diagnosed by Hepato-gastro-intestinal pediatricians because the intractable diarrhea or the liver disease are the foremost signs. The main differential diagnoses are other causes of chronic diarrhea. Most of the non-genetic causes (infections and allergies) can be ruled out by explorations and clinical observation as they are resolved with adequate treatment. Among intractable diarrhea of infancy, only the syndromic tufting entheropathy also presents hair abnormalities [19], but it presents also specific pathologic abnormalities and can easily be differentiated by histological investigations. Children suffering from severe malnutrition can have hair abnormalities due to copper or iron deficiency but these features resolve with renutrition. In rare cases, neurofibromatosis has been evocated because of numerous café au lait spots.

## Genetic counseling

SD/THE is an autosomal recessive disorder. Considering the severity of the disease, an antenatal diagnosis could be offered to the parents of an affected child once the molecular defect is characterized.

## Management including treatment

The management concerns the three life-threatening signs: intractable diarrhea leading to failure to thrive, immune abnormalities and liver disease. Intractable diarrhea often needs a parenteral nutrition to achieve nutrition and growth, during a variable period of time ranging from a few months to several years. In some cases, the association of enteral with parenteral nutrition can be indicated. For reasons of immune disorders, immunoglobulin supplementation is sometimes necessary and the antibody production after vaccination should be monitored. Finally, some children can present a severe liver disease independent of parenteral nutrition [2,6,12] which could be worsened by the parenteral nutrition. In case of severe liver disease, the only therapeutic option is liver graft.

## Prognosis

Even if the prognosis seems to have improved with years, there is still a high mortality rate (3/12 in Hartley et al. 2/12 for Fabre et al. and personal data). The main complications are liver disease and infections. Some patients are weaned off parenteral nutrition [2,13] but others remain under parenteral dependence for more than 10 years. Most of the children achieve a small final stature and half present a slight mental retardation.

## Unresolved questions

The discovery of the molecular basis of SD/THE opens the perspective to better understand the disease and indicates that it must be considered as a mRNAs quality control disease even though the link between mutations in *SKIV2L* or *TTC37* and dysfunction of the Ski complex remains to be formally demonstrated. Further investigations will allow us to understand how the alteration of a basic function such as RNA decay leads to these specific signs. There are probably several ways and environmental interactions which have to be investigated and a part of the pathology could be caused by misregulation of non coding RNAs. The possibility of the existence of milder forms, especially regarding gastrointestinal signs, has to be considered. In 2008, Al Qoer et al. described a cohort of children with mild diarrhea, hair and skin abnormalities who may possibly present an authentic SD/THE [20].

WDR61, the third component of the SKI complex, would have been a candidate gene for SD/THE in patients with no mutation in *TTC37* or *SKIV2L*, but it supports other functions in meiosis and in the PAF complex making its involvement improbable [21].

## Conclusion

SD/THE is the first mendelian disease linked to a cytoplasmic exosome anomaly. With at least 2 genes involved, it is a genetically heterogeneous disease which should be evocated in cases of intractable diarrhea with hair abnormalities and confirmed by molecular diagnosis. Although improvements have been made recently regarding molecular basis and diagnosis, the prognosis remains severe.

## Competing interests

The author declares no competing interests.

## Authors’ contributions

AF and CB take responsibility for the review and have been involved in drafting the manuscript and revising it critically; AF, CMV and OG conducted clinical investigations, collected clinical data and revised the manuscript. All authors have given final approval of the version to be published.

## Consent

Written informed consent was obtained from the patient’s parent for publication of this report and any accompanying images.
